# The ontological inversion: transcending adultcentrism in the concept of development for the critical deconstruction of bleak pedagogy

**DOI:** 10.3389/fpsyg.2026.1794220

**Published:** 2026-03-19

**Authors:** Yao Luo, Xi Yang

**Affiliations:** School of Education, Hunan Normal University, Changsha, China

**Keywords:** adultcentrism, bleak pedagogy, development, intersubjectivity, ontological inversion, process-relational becoming

## Abstract

Traditional developmental psychology is increasingly critiqued for its adultcentric bias, which conceptualizes childhood as a state of functional deficit and a mere transition toward the teleological actuality of adulthood. This ideological framework provides a scientific sanctuary for bleak pedagogy—a repressive educational paradigm characterized by will-breaking and corrective violence. This paper conducts a meta-theoretical deconstruction and reconstruction of the concept of development by tracing its philosophical inheritance from Aristotelian entelecheia through Enlightenment rationalism to modern Darwinian linear evolution. By exposing how these narratives legitimize the systematic marginalization of children’s current agency, the study proposes an ontological inversion. It shifts the developmental paradigm from a linear, bio-reductionist “ladder” toward a Relational Developmental Systems (RDS) framework that emphasizes Aionian presentness and situational becoming. Under this lens, development is reimagined as a non-linear, intersubjective process of co-becoming, where the child is no longer an incomplete subject in need of repair but an active social agent within a reciprocal context. By establishing a relational pedagogical paradigm, this research dismantles the operational pillars of bleak pedagogy—future-investment instrumentalism and the tyranny of developmental norms. It concludes that the holding environment restores the ontological dignity of childhood, transforming education from a standardized production line into a cradle for vital diversity and the flourishing of life.

## 1 Introduction

The concept of development constitutes the foundational theoretical paradigm for understanding, measuring, and intervening in the life courses of children (Morss, 1996; Burman, 2008). Within developmental psychology and educational research, its theoretical constructs—including fixed milestones, stage theories, and deficit models—exert a dominant influence on curriculum design, clinical assessment, and social policy (Stone, 1996; Gabriel, 2021). Given its high utility and seemingly scientifically neutral authority, this paradigm profoundly shapes how adults perceive the child, particularly concerning normative expectations and deviations from the mean (Fuller, 2007; Hamar et al., 2025). As noted by critical developmentalists, these normative frameworks often function as a restrictive lens that prioritizes universal trajectories over individual or cultural variations (Morss, 1990; Lerner, 2018).

However, this paper argues that the widespread acceptance of the development concept masks deep-seated theoretical and ethical instabilities. Through a conceptual history analysis (Section 2), we reveal that development is not an objective measure of maturation, but a construct systematically encoded by philosophical teleology and reinforced by the linear logic of modern evolutionary models (Gesell, 1939; Harris, 1957). This historical encoding invariably posits the mature adult state as the ideal terminus (*Telos*), thereby constituting the core ideological anchor of this study: adultcentrism (Petr, 1992).

Crucially, this adult-centric paradigm manifests in the “deficit conception of childhood,” which negates the child’s intrinsic ontological value and legitimizes the repressive practices of what we term bleak pedagogy (Section 3; Florio et al., 2020b). To address this fundamental instability, this paper contends that developmental theory must move beyond descriptive models to confront the ontological questions of the child’s being (Kennedy, 2006; Murris, 2016). This necessitates an ontological inversion—a radical shift from prescribed ladders of progress to a paradigm of process-relational becoming (Section 4; Overton, 2015).

### 1.1 Research aims and structure

This study aims to conduct a meta-theoretical deconstruction of the adult-centric development concept and propose a robust philosophical foundation for a relational and emancipatory educational paradigm (Morss, 1996). Specifically, we address the following aims:

To trace the conceptual history (Section 2) of “Development,” revealing how Aristotelian teleology and linear evolutionary models established the adult as the universal benchmark, thereby creating the deficit conception of childhood (Matthews, 1994; Kennedy, 2006).

To critically examine the structural mechanisms of bleak pedagogy (Section 3), demonstrating how the dominance of the development paradigm leads to the tyranny of norms, the instrumentalism of future-investment, and the systematic erasure of the child’s agency (Rutschky, 1977; Florio et al., 2020b).

To propose the “Ontological Inversion” framework (Section 4), reconstructing development as a non-linear, situational, and relational flow (*Aion*) rather than a prescribed climb toward adulthood (*Chronos*) (Kohan, 2011; Murris, 2016).

To establish the Relational Pedagogical Paradigm, outlining the shift from adult-centric dominance to a “Holding Environment” characterized by intersubjective encounter and co-becoming (Overton, 2015).

The paper is structured as follows:

Section 1: The Historical Benchmark analyzes the conceptual history of development and the formation of the deficit conception of childhood.

Section 2: Structural Impact examines the ontological crisis, the will-breaking practices, and the instrumentalism resulting from the dominance of the development concept.

Section 3: Paradigm Reconstruction proposes the framework of ontological inversion and outlines a new educational ethics based on process-relational becoming.

Section 4: Conclusion summarizes the theoretical contributions and discusses the implications for future empirical research.

## 2 The historical benchmark: the adult-centric shaping of the concept of development

The critical deconstruction of the concept of development must be anchored in a rigorous examination of its conceptual history, which is the necessary path for addressing its persistent theoretical and practical controversies. As this study’s introduction emphasizes, development—though a core concept in contemporary childhood discourse—is marked by extensive influence and high usage while simultaneously concealing numerous theoretical disputes and practical ambiguities. This complexity was eloquently captured by Harris (1957), who noted that the concept of development has impressed scholars as being slippery and of limited usefulness, with few attempting to master it in its many aspects.

### 2.1 The theoretical imperative and foundational thesis

This structural ambiguity is not accidental; its root lies in the theoretical nature of the concept of development, rather than its empirical status. Lerner (2002) articulates this point: development is not an empirical concept. As he emphasizes, “One’s study of development begins with some implicit or explicit concept of what development is” (p. 16). This premise forms the bedrock of this study’s core argument: the empirical investigation of childhood development is invariably preceded by a theoretical understanding of development itself. Furthermore, how we define the human condition fundamentally dictates our understanding of development and the child, which subsequently determines the ethical and structural parameters of education.

It is precisely because of the theoretical nature of development that its historical construction, from its inception, was inevitably subject to the profound influence of its social-historical context. This social-historical context was not value-neutral; rather, it was pre-dominated by the discourse and power structures of adult society. Consequently, the concept of development, throughout its construction and evolution, systematically and structurally prioritized the encoding of the adult state, thereby directly shaping and cementing the ideological foundations of Adultism. To thoroughly reveal the structural privilege of the adult, which is deeply rooted in this social-historical context, and to analyze its mechanisms, we must trace the two core stages in which the concept of development was encoded: the philosophical inheritance of teleology and the scientific reinforcement of evolutionism.

### 2.2 The philosophical inheritance of teleology (*Télos* and *Entelecheia*)

The dominance of the modern concept of development lies in its inheritance of the philosophical core of adultcentrism: Teleology. In the pre-modern context, the teleological understanding of change was the prevalent paradigm, with its most representative expression found in Aristotle’s concept of *Entelecheia* (or *Télos*). This concept posits that every entity contains an internal, ultimate goal, rooted within the framework of potentiality (*dynamis*) and actuality (*energeia*). According to Aristotle c. 350 B.C.E. (1998), *entelecheia* represents a complete reality—the state where a thing has realized its inherent *telos*. Etymologically, Aristotle constructed the term by combining *entelēs* (ἐντελής, “complete”) with *echein* (to have/hold), suggesting a state of “having one’s end within” (Sachs, 1995; Reeve, 2018).

Within this framework, the change of an entity is viewed as the unfolding of a potential seed inherent in its essence, directed toward its own internal, ultimate purpose. As Aristotle c. 350 B.C.E. (1998) defines it in *Physics* Book III, motion itself is “the complete reality of what exists potentially, insofar as it is such” (201a10). This implies that *entelecheia* is not a static endpoint but a dynamic state of being fully what one is meant to be. This teleological perspective exerted a profound influence, originating in Greek thought and becoming further systematized through Medieval theology, eventually providing the metaphysical template for the modern ladder of development, where the child’s value is perpetually measured against the complete reality of the adult.

During the Enlightenment, although traditional religious teleology faced challenges, this teleological outlook persisted. Leibniz (1714/1898, p. 244) reinterpreted *Entelecheia* through his theory of Monads, deducing that: “In God there is Power, which is the source of all, also Knowledge, whose content is the variety of the ideas, and finally Will, which makes changes or products according to the principle of the best” (Leibniz, 1714/1898, p. 244). This fundamentally teleological worldview posits that change is inherently oriented toward perfection.

Hegel (1822–1831/1956, p. 38) subsequently brought this tradition to its philosophical peak, defining Development (*Entwicklung*) as the ultimate trajectory of the Absolute Spirit to realize its full potential—a movement from the in-itself (*an sich*) to the for-itself (*für sich*). This grand dialectical vision asserts that the Spirit’s latent capacity must transition into an external, self-conscious reality: “the truly good—the universal divine reason—is not a mere abstraction, but a vital principle capable of realising itself”. Thus, within Hegel’s philosophical structure, the historical process itself is defined as the necessary and complete unfolding of the Spirit toward its ultimate purpose and freedom.

This metaphysical principle was readily mapped onto the human life course, transforming the Aristotelian *dynamis/energeia* framework into a moral and ontological hierarchy. It is precisely this philosophical transfer that provided the initial ontological legitimacy for adult privilege: the mature adult state, endowed with Enlightenment ideals of rationality and autonomy, was framed as the paradigm of actuality (*energeia*) and perfection (*Télos*). Conversely, the child was relegated to a state of pure potentiality (*dynamis*). This encoding designated the child’s existence as a mere process of becoming, fundamentally denying their ontological completeness in the present. This teleological legacy constructed the foundational logic of adultcentrism: the child’s value is perpetually postponed, and their existential meaning is defined solely as an investment in the future adult state.

### 2.3 The scientific reinforcement: evolutionary linear logic

Teleology served as the core framework for interpreting change long before development emerged as a standalone concept. The radical shift occurred with the rise of scientific discourse, primarily driven by Darwinian evolutionary theory. This scientific impetus was intended to enact a rupture with traditional teleological contexts, aiming for a secular, non-teleological understanding of progression.

However, this intended break ultimately failed to escape the shadow of its philosophical predecessor. While the modern concept of development initially attempted to de-teleologize the trajectory of human progress, the linear logic of evolution effectively provided a scientific endorsement of the original teleological structure. This reinforcement transformed adult privilege from a philosophical metaphor into a set of rigorous, institutionalized normative mechanisms.

The ideological shift of the concept of development corresponds to a distinctive linguistic change of the modern era, which Koselleck termed the emergence of the “collective singular” (*Sammelbegriff*). This transition attempted a rupture with tradition, but its incorporation into a scientific framework performed a secular inheritance by embedding a singular, fixed direction of progress into the language of science. As Koselleck (1979/1985) notes, in the decades around 1800, concepts like “Progress” and “Development” began to contain “temporal indications that had never before been used in the same way” (p. 271).

During the 18th and 19th centuries, the terms *progress, development,* and *evolution* held no significant semantic differences. The word *evolution* originated from the Latin *evolutio(n-)*, meaning “unrolling,” derived from the verb *evolvere*. Early senses related to physical movement, such as a tactical wheeling maneuver of troops, but it eventually evolved to denote “opening out” or “unfolding” (Harper, 2024). The popularization of *evolution* is most directly tied to Darwin’s (1859) *On the Origin of Species*, where he used the verb *evolve* to conclude his monumental work: “beginning endless forms most beautiful and most wonderful have been, and are being, evolved.” (p. 491).

Against the backdrop of 19th-century scientism, development became synonymous with evolution. As Harris (1957) noted, “The concept of development is fundamentally biological. Commonly associated with the organization of living structures and life processes” (pp. 3–14). This concept was further standardized in the mid-20th century into a scientific consensus, framing development as a “sequential set of changes” within a system of “pre-existing capacities” (Nagel, 1957, pp. 15–24). By establishing development as a fixed, sequential, and structurally pre-determined process, this scientific consensus transformed an abstract biological principle into a universally applicable normative scale. This neutral language effectively provided the objective mandate necessary to enforce a societal hierarchy, where the child is measured against the adult benchmark of functional completion.

### 2.4 Institutionalization, normative tools, and deficit conception of childhood

The institutionalization of the developmental benchmark was deeply entwined with the cultural history of childhood. During the 19th century, European Romanticism and American Transcendentalism shifted the narrative from “original sin” to “innate innocence” (Rousseau (1762/1979), laying the ethical groundwork for Progressive Education. While these movements championed child-centeredness, they paradoxically reinforced the child’s status as a natural but incomplete being (James and Prout, 1997). This cultural ideal of maturation defining the endpoint of human progress—the mature adult state—as the highest and most adaptive stage.

This transition granted adult-centric legitimacy, and provided the essential scaffolding for Developmental Psychology. Its founder, G. Stanley Hall, integrated into individual development, prioritizing objective, rational, and empirical scientific research methods to identify the universal laws of child development (O’Donnell, 1985). The influence of Darwinian thought on child development was further solidified by Gesell (1939), pp. 548–553 who emphasized the crucial role of biological factors and “maturational readiness”. This evolutionary view also permeated early theories of cultural progression through anthropological thought. For instance, Tylor (1871) viewed social evolution similarly to biological organisms, arguing: “the savage state in some measure represents an early condition of mankind, out of which the higher culture has gradually been developed or evolved progress has far prevailed over relapse” (p. 28).

The interpretation of childhood from a stagewise perspective became the dominant paradigm in 20th-century child psychology, represented by Freud, Erikson, Gesell, and Piaget. These theorists posited that child development follows a process analogous to evolution, where later stages supersede earlier ones due to their greater adaptation to reality. Piaget’s (1932) description of the child’s sense of justice serves as a classic example, where justice is first subordinated to adult authority before evolving into progressive equalitarianism and equity (p. 314).

Damon and Lerner (2006) argue that if a prevailing metatheory asserts the legitimacy of transformational change, then theories of development will include some type of “stage,” “phase,” or “level” (p. 21). Under the dominion of this evolutionary linear logic, developmental milestones and stage theories transformed into normative tools possessing a veneer of objectivity. Any child who deviates from the adult-prescribed linear path is diagnosed as developmentally delayed, a scientific diagnosis that systematically produces deficiency.

The combination of these scientific mechanisms solidified the ideology known as the deficit conception of childhood—the explicit ideological manifestation of structural adultcentrism. Matthews (2008) identifies this model, noting that under its influence, society operates on the assumption that children “are well thought of primarily as lacking certain competencies that they can expect to develop by the time they are adults. Are essentially incomplete human beings.” This model negates the child’s present existence, stripping it of intrinsic ontological value and degrading its identity to a mere tool for the future needs of adult society. This negation drives Bleak Pedagogy (Section 2) and justifies the ontological inversion advocated by this research (Section 3).

## 3 Structural impact: the adult-centric view of development as the ontological foundation and practical mechanisms of bleak pedagogy

Section 1 revealed the concept of development as an adult-centric ideological framework, culminating in the deficit conception of childhood. The genuine danger of this encoding lies in its capacity to function as a structural power. The concrete manifestation of this power is Bleak Pedagogy (BP). Originating from Rutschky's (1977) foundational work, *Schwarze Pädagogik* (Black or Bleak Pedagogy), BP is a disciplinary approach rooted in adult authority and control, often involving punitive methods perceived as culturally acceptable (Florio et al., 2020b, 2022). Section 2 bridges the gap between ideology and practice, analyzing how this adult-centric view provides the ontological foundation and instrumental mechanisms for BP, ultimately necessitating an ontological inversion.

### 3.1 The ontological precondition of bleak pedagogy: how deficit encoding legitimizes adult dominance

The existence of Bleak Pedagogy rests upon a profound ontological precondition: the deficit encoding inherent in the adult-centric view of development. By classifying the child as incomplete potentiality (dynamis), this encoding grants absolute dominance both moral and scientific legitimacy.

#### 3.1.1 The core encoding: the ontological designation of the child as an incomplete subject

This section contends that the adult-centric view of development enacts a systematic ontological deficit encoding upon childhood subjectivity. This encoding is not merely descriptive but profoundly normative, stemming from a deep-seated teleological structure that designates the child’s existence as inherently incomplete and incompetent (Petr, 1992).

The genesis of this deficit encoding lies in the governing paradigm of Adultcentrism (AD). Petr (1992) defines AD as the tendency of adults to view children and their problems from a biased, adult perspective—a bias that is a structural and cultural norm rather than an accidental individual error. This paradigm functions by establishing the adult as the *Télos* (endpoint) and actuality of development, thereby confining the child to the precarious categories of becoming and potency (Németh et al., 2024).

The core bias of adult-centric stage theories functionally defers the child’s full value and significance to a future adult stage. As Biswas et al. (2024a, 2024b) critically argue, this process reinforces an adultist hierarchy, viewing children as incomplete beings in a preparatory phase called childhood. This ontological setup has devastating consequences for power dynamics: it not only negates the child’s present subjective agency but also structurally strips them of their right to participate as epistemic agents. Once the child is encoded as deficient, the adult’s exertion of disproportionate intervention and dominance ceases to be seen as an abuse of power; instead, it is reframed as a moral responsibility and a scientific necessity.

#### 3.1.2 The power transfer: the logical leap from developmental guidance to absolute dominance

Deficit encoding converts the adult’s responsibility to guide into a mandate for absolute dominance. This transfer provides unassailable legitimacy for the practices of Bleak Pedagogy. The unidirectional nature of development grants the “higher” level (adult) teleological pre-authorization over the “lower” level (child), negating alternative paths for their becoming (Nodelman, 1985). By standardizing developmental stages, the adult assumes the status of a developmental expert. This expertise creates a sense of distance and superiority, ultimately leading to adultist discrimination (Matthews, 1994). This expert-patient model structurally transforms the responsibility to guide development into an unconstrained power of intervention.

This expert authority is realized through the mechanism of objectification, resulting in the alienation of the child’s identity. Developmentalism positions the child as an objectively graspable object of study. As Morss (1996) notes: “Development is objectified: it becomes solid and measurable, a field of study and of expertise. Instead of seeing people we see people developing” (p. ix). Within this professional framework, the child is seen as an object to be measured, compared, controlled and actively formed. In other words, the child is positioned as a lowercase “i” (an individual object), while the adult researchers occupy the uppercase “I” (a fully-formed human subject) (Murris, 2016).

The adult-centric nature of knowledge production further solidifies this power loop. This translates the ethos of guidance into the reality of dominance, completing the power cycle: (1) the adult secures ontological legitimacy through deficit encoding; (2) achieves power transfer through expert status; and (3) institutionalizes Bleak Pedagogy, defined by adult authority and control (Florio et al., 2022). Thus, the adult-centric view of development is the core structural driver enabling the normalization of BP.

### 3.2 The operational logic of bleak pedagogy: instrumentalism and the tyranny of developmental norms

If Adultcentrism provides the ideological blueprint for this unequal intergenerational relationship, then Bleak Pedagogy serves as its architectural execution (Florio, 2018). By weaponizing teleological and evolutionary logic, BP transforms the child into a site of instrumental investment and normative correction. Consequently, education is reduced to a disciplinary project aimed at aligning children with adult-predefined trajectories. This process functions through two mechanisms: teleological future-investment and the evolutionary tyranny of norms.

#### 3.2.1 Mechanism I: the instrumentalism of future-investment (teleological practice)

The primary operational mechanism of BP lies in its instrumental objectives, which treat education as a future-investment to achieve predefined social and individual ideals. Florio (2018) and Florio et al. (2020a, 2020b) defines adultcentrism as a cognitive bias that positions the adult as the Télos, reducing the child to a state of being not-yet-complete. Within this paradigm, children are trapped in a deficit conception of childhood where their value is measured solely by functional lack. This reinforces the value orientation criticized by Gheaus (2018), wherein contemporary theories prioritize the future well-being of the adult over the present flourishing of the child. By categorizing children as mere immature versions of rational beings (Burman, 2008), this teleological perspective systematically hollows out their subjective status during the protracted phase of child-becoming-adult.

This conceptual framework translates into a stark practical objective: the systematic breaking and reshaping of the child’s will. Rooted in a deep-seated mistrust of children’s inherent capabilities (Florio et al., 2020a, 2020b), BP views childhood as a mere transitional life form (Kennedy, 2006). Consequently, any manifestation of a child’s agency that deviates from adult-predefined trajectories is perceived as an obstacle to development. This directly drives the extreme measures described by Hamar et al. (2025): the essence of black pedagogy is the systematic application of methods aimed at “breaking the will” to mold character according to the idealistic values of adults or society. Here, the child’s present is treated as inefficient raw material that must be disciplined to produce a valuable future.

The ultimate culmination of this mechanism is a systemic negation of the child’s subjective value in the here and now. As Kohan (2011), argues, education ceases to be the flourishing of life and becomes a tool for realizing adult social ideals. Xiong (2008) profoundly captured this teleological temperament, noting that when life is believed to have a fixed functionality, childhood itself is rendered devoid of intrinsic meaning. This perspective provides BP with moral justification to plunder the child’s present under the guise of benevolence. Cloaked in manipulative care, educators utilize disproportionate authority (Florio et al., 2022) to prune childhood traits deemed functionally irrelevant. This future-investment thus becomes an alienated contract: the child must surrender their current agency in exchange for a future identity validated by adults.

#### 3.2.2 Mechanism II: the tyranny of norms (evolutionary practice)

While teleology defines the end goal, the evolutionary logic of linear stages provides BP with a rigid yardstick for measurement and regulation. This mechanism transforms adult-centric dominance into a technically rationalized disciplinary project by scientificizing development into a set of objective norms.

The logic of this tyranny is fundamentally rooted in the weaponization of Developmentalism. Heavily influenced by evolutionary natural selection, this view presupposes a natural course and universal laws of growth (Morss, 1996). When adulthood is positioned as the absolute apex of evolution, any child whose performance deviates from the predefined linear trajectory is scientifically diagnosed as a deficit. Florio (2018) points out that this bias is particularly evident in the surge of Specific Learning Disorder (SLD) diagnoses, where educators use medical labels to objectify a child’s heterogeneity. As Vandenbroeck (2006) reveals, norm-based assessments often act as a mechanism of social exclusion, wherein scientific standards are utilized to pathologize diversity and categorize children into those who are normal and those who require expert intervention.

Moving from assessment to intervention, the linear logic of development drives a pathological intolerance of errors. Under the adult-centric gaze, development is reduced to a rigid timetable; any minor lag is treated as an ontological deficit or a risk of derailment from the adult-defined Télos. This supports the obsession with correctivity identified by Florio et al. (2020b). BP operates on the steadfast belief that every error or disobedience must be met with corrective measures, a stance rooted in fundamental mistrust. Hamar et al. (2025) further expose the darker side of this logic, arguing that when educational norms are institutionalized, they provide adults with a scientific mandate to suppress any behavioral deviation, justifying the erasure of the child’s individuality as a necessary step toward normative progress.

This trajectory culminates in the normalization of surveillance and the exercise of disproportionate authority. By naturalizing the life course into measurable data (Morss, 1996), BP grants educators the privilege of all-encompassing surveillance. Florio (2018) indicates that adultcentrism often leads adults to utilize their status to impose authority that is vastly out of proportion to the child’s needs. Within the BP framework, this manifests as manipulative educational behavior. As criticized by Miller (1983), adults exert a form of hidden cruelty through pedagogical manipulation, systematically breaking the child’s will under the guise of benevolence. Ultimately, this repressive upbringing dissolves the child’s vibrant spontaneity, transforming their unique life trajectory into a compliant product of social adaptation.

### 3.3 The critical predicament: why current theories fail and the necessity of ontological inversion

Undeniably, ethical critiques of “future exploitation” (Gheaus, 2018), psychological reflections on “disproportionate authority” (Florio, 2018), and empirical analyses of the conflict between legal norms and educational practices (Hamar et al., 2025) have provided essential weapons against BP. These critiques have successfully exposed moral deficits and power violations, curbing overt violence. Nevertheless, BP practices persist tenaciously within the micro-textures of modern education. This critical predicament stems from a deep-seated defensive mechanism: BP is not merely a failure of methods, but a power logic parasitic upon the modern concept of development.

In previous academic discourse, development has been treated as a neutral fact, failing to recognize its role as the ideological core of Adultcentrism. It is teleology that locks the child into the status of incomplete, and evolutionary logic that reduces life to a standardized ladder. As long as this definition remains in adult hands, BP can continually legitimize the crushing of the will under the scientific guise of facilitating progress. The limitation of existing theories lies in their failure to shake the “deficit encoding” embedded within the concept of development itself.

To thoroughly dismantle the legitimacy of BP, one must achieve a leap from criticizing manifestations to criticizing the underlying paradigm. The linear, stage-based concept of development is the ultimate sanctuary for BP practice. This realization necessitates an Ontological Inversion: liberating the child from the evolutionary process toward adulthood (child-becoming-adult). This inversion involves a fundamental reconstruction of development itself. Within this new horizon, development is no longer a skill accumulation aimed at adulthood, but a continuous self-unfolding of life. Childhood is no longer a functional deficiency, but a unique state of being that holds intrinsic meaning. This leads directly to our subsequent reconstruction: establishing a new pedagogical paradigm that is neither adult-centric nor premised on deficit.

## 4 Beyond developmentalism: the ontological inversion of development and the reconstruction of the relational pedagogical paradigm

The tenacity of Bleak Pedagogy is an ontological consequence of the adult-centric construct of development. To challenge this foundation, Section 3 initiates an ontological inversion, reimagining development not as a tool for disciplining the future, but as a relational practice of co-becoming.

### 4.1 Discursive deconstruction: developmentalism as the ideological mask of adultcentrism

Developmentalism, as the ideological mask of adultcentrism, encapsulates modernist claims regarding the naturalness, universality, and teleology of human growth. At its core, this paradigm relies on the essentialist assumptions of naturalness and universality. James and Prout (1997) identified the three pillars of modernist childhood studies as “rationality”, “naturalness”, and “universality”. Developmentalism fully embodies these traits; influenced by evolutionary natural selection, it advocates for a natural course of child development. As Stone (1996) argues, developmentalism presumes typical patterns of change to be optimal simply because they are natural. This perspective suggests that the individual child’s psyche is formed according to its innate potential (Murris, 2016). Paradoxically, by ostensibly curbing adult interference to protect this natural course, developmentalism establishes an even more insidious form of dominance: the power to define nature itself. By transmuting adult-prescribed values into immutable natural laws, this paradigm legitimizes adult authority under a biological cover.

From a postmodern perspective, this pursuit of essence is illusory. Deleuze and Guattari (1980/1987) posits that “there is no beginning or end to rhizomatic lines. They are always in the middle of dynamic movement; hence they form multiplicities that change in character. Lacking any identity or essence” (Best and Kellner, 1991, p. 100). Similarly, Derrida (1972/1981) argues in Positions that there is no transcendental subject who acts as the “master” of development; rather, subjectivity is merely an “effect of *différance*” (p. 28). By establishing a Darwinian myth, it situates children at the bottom of a civilizational ladder. As Kennedy (2013) concludes, this critique of developmentalist practice as an expression of “colonizing biopower” marks the “negative space” from which postmodern discourses of childhood emerge (p. 146).

Crucially, the power of this biopower is sustained by a temporal hegemony of teleology and progressivism. While some attempt to distance evolutionary development from teleology, postmodernists argue that developmentalism persists as a teleological expression that inevitably orients the child toward a specific, predetermined end-state: adulthood. This belief in progress frames human development as a hierarchical ascent. This linear order is what Deleuze and Guattari (1980/1987) critique as “arborescent (tree-like) multiplicities”—hierarchical systems that “always presuppose a fundamental, powerful unity” (p. 5). In this context, the idealized adult subject serves as the unity or pivot-root that organizes and subordinates all diverse paths of childhood becoming.

To dismantle this dominance, it is not enough to acknowledge the child’s perspective; rather, as Derrida (1972/1981) asserts, “to deconstruct the opposition is to overturn the hierarchy at a given moment” (p. 41). Burman (2008) unmasks the colonial logic embedded within this framework, identifying developmentalism as a hallmark of nineteenth-century European thinking where children, women, and “primitive” peoples are marginalized as “immature versions of the adult, male, rational mind” (p. 11). When adultcentrism dictates a predetermined destination for growth, the very concept of liberty is compromised. As Morss (1996) provocatively argues, the so-called “freedom to develop” is, in fact, a “freedom with strings attached”—a contractual trap (p. 9). Ultimately, developmentalism is radically antithetical to human freedom, subjecting the individual to a prescriptive trajectory serving adult-centric control.

This discursive deconstruction reveals that developmentalism is not an objective scientific truth, but a cultural discourse and a political technology. To terminate this power logic, we must shatter the false rhetoric of the not-yet-adult and replace teleological lack with a radical sense of becoming. We must transition from “what the child will be” to an ontological re-examination of “who the child is,” restoring the relationship between adult and child to one of mutual, non-hierarchical co-becoming.

### 4.2 Reconstructing development: from prescribed ladders to the process-relational becoming

The reconstruction of development moves beyond milestone schedules toward a comprehensive ontological paradigm shift. To revoke deficit encoding, the child’s life is reconfigured across three dimensions: Time: De-teleologization through the lens of *Aion*, liberating growth from the linear shackle of “becoming-adult.” Space: A contextual turn that dismantles the myth of biological reductionism. Dynamics: A relational reshaping that establishes the logic of process-relational co-becoming.

#### 4.2.1 The de-teleology of development: from the discipline of *Chronos* to the becoming of *Aion*

The primary task of reconstructing development is to excise the toxins of linear progressivism. Traditional developmentalism, influenced by Aristotelian *Entelecheia*, defines the child as a site of lack or a transitional state. This logic imprisons the child within the oppression of *Chronos*—a quantitative, segmented, and programmed linear sequence that mandates life to meet milestones at prescribed intervals. Under this disciplinary temporality, child development is reduced to a debt toward adulthood, where the intrinsic meaning of the present is perpetually overdrawn by the looming standards of future maturity.

To resist this temporal violence, this study advocates for an ontological shift toward *Aion*. Within the horizon of *Aion*, time is no longer a container for objects but a plane of pure becoming. Unlike the successive presents of *Chronos*, *Aion* stretches the moment into an infinite past and future, rendering development not as a spatial displacement from point A to point B (child to adult), but as the intensive eruption and differentiation of life in its virtuality. Since Heraclitus (2001), *Aion* has been inextricably linked to childhood and play: “*Aion* is a child at play, playing draughts; the kingship is a child’s” (Heraclitus, Fragment B52). In this Heraclitean vision, the sovereign power of the cosmos resides not in adult-like order or *telos*, but in the non-purposive play of becoming. Deleuze and Guattari (1980/1987) deepened this tradition, asserting that “a line of becoming has neither beginning nor end. Neither origin nor destination” (p. 293). Within this de-teleological horizon, childhood (*infantia*) is re-envisioned as a form of “Virtual Mastery” (Kennedy, 2024, p. 53)—a “Wild Being” that resists the external apparatus of control that seeks to bind *Aion* into the segments of *Chronos*.

Within this framework, “Becoming-child” emerges as a subversive force. All becoming is, in essence, a becoming-minor, seeking not the molar pinnacle of power but the molecular overflow of life. As Kennedy (2013) notes, “Becoming-child is a line of flight, a trajectory of escape from the subject identified by law, custom, and subjectivity itself” (p. 146). This flight shatters the continuity of *Chronos* through *Kairós*—the opportune, qualitative moment that “opens a space for an encounter with *Aion*” (Kennedy and Kohan, 2008). Furthermore, “Becoming-child” is not a physiological regression but a movement of intensity. As Kohan (2011) emphasizes, it is a “revolutionary space of transformation” where the subject occupies a flux of change rather than a fixed age. When development is reconstructed as an *Aion*-based becoming, it no longer requires the mature adult state as its teleological coordinate. Consequently, child and adult achieve ontological coexistence, shifting the focus of development from approximating a predetermined end to the infinite differentiation of life intensity, thereby completing the deconstruction of the adult-centric binary opposition.

#### 4.2.2 The spatial reconstruction of development: from biological reductionism to situational becoming

Following the temporal de-teleologization, the second dimension of reconstruction involves a spatial expansion—shifting from closed, biologically reductionist individualism toward a dynamic contextualism. This paradigm shift asserts that development is no longer a pre-programmed natural blueprint; instead, context must be elevated to an ontological core. Within this reconstruction, the term context is prioritized over environment to emphasize the fluid complexity and the continuous, reciprocal interaction between the child and the world.

This spatial reconstruction is driven by a synthesis of internal psychological progress and external interdisciplinary critiques. Bronfenbrenner (1977) famously challenged decontextualized laboratory experiments, labeling them “the science of the strange behavior of children in strange situations with strange adults for the briefest possible periods of time” (p. 513). He advocated for an “experimental ecology” where development is viewed as a nested system of interconnected layers. From this perspective, psychological functions are no longer isolated biological attributes. As Jenkins (1974) argued, psychological phenomena cannot be explicated by reduction into atomistic units; rather, their essence is manifested only within the quality of particular event contexts. This context-dependency anchors Lerner’s (2002) concept of “lifelong plasticity,” which reimagines development as a product of continuous person-context reciprocity.

Concurrently, interdisciplinary childhood studies have dismantled the psychological monopoly over development. Historians and sociologists argue that while immaturity is a biological fact, childhood is a socio-cultural construct Ariès (1960/1962). As Postman (1982) contended, although childhood possesses a biological basis, it cannot be realized unless a social environment triggers and nurtures it. The New Sociology of Childhood further maintains that the ways in which biological immaturity is made meaningful is a “fact of culture” (Prout, 2005, p. 55). Anthropologist Lancy (2015) exercises “the anthropologist’s veto” to critique the ethnocentric nature of developmental psychology, using ethnographic studies to “de-universalize” stage theories that ignore cultural particularity.

In the realm of education, the integration of Dewey’s “experience” and Vygotsky’s “socio-cultural context” has formalized the necessity of situated development within diverse ecological systems (Burman, 2008; Lerner, 2018). By shifting focus from predetermined biological structures to the process of change, development is reimagined as an *Aion*-based trajectory shaped by diversified contexts. This reconstruction restores the agency of children as active social agents and affirms the inherent expansive possibilities within their developmental paths.

#### 4.2.3 The relational ontological reshaping of dynamics: from individual attributes to RDS-based “co-becoming”

The final dimension of reconstruction involves a fundamental shift in underlying dynamics—moving from the substantialism of individual attributes toward Relational Ontology and Relational Developmental Systems (RDS) theory.

The reshaping of developmental dynamics begins with a robust critique of developmentalism. As Gabriel (2021) persuasively argues, traditional psychology’s adherence to universal stages functions more as a tool for social control than as an explanation of growth. He advocates for a “relational approach” that reimagines development as a “non-linear, temporal, and embodied process” (p. 48).

This shift from “substance” to “process” finds its philosophical bedrock in the work of Overton (2015). Overton critiques the “Cartesian Split”—the modernist tendency to treat nature/nurture or subject/object as independent variables. In its place, he proposes a “Process-Relational Worldview,” asserting that the motor of development resides within the indissoluble fusion of the individual and the environment. This is further supported by Tomasello (2019), who contends that the unique driver of human development is “Shared Intentionality”—the evolved urge to coordinate intentions and engage in collaborative activities. Consequently, human cognition and morality are not pre-wired to mature in isolation; rather, they are activated through ongoing intersubjective negotiation.

At the level of identity, this dynamic manifests as the “dialogical self.” Taylor (1991) reminds us that identity depends on “dialogical relations with others” (p. 48). Expanding on this, Kennedy (2024) draws on Dialogical Self Theory to describe the self as a dynamic space comprising multiple “I-positions.” Developmental dynamics are thus reimagined as continuous “boundary-crossings” between internal narrative voices and external social others.

Ultimately, Lerner (2018) integrates these perspectives within the RDS framework, defining development as a process of co-becoming. Within this system, experience is a transformative fusion of the person and their multifaceted context. This reconstruction liberates developmental dynamics from isolated biological engines, repositioning it as a relational force exercised within intersubjective spaces. Accordingly, the educational environment is transformed into a “Holding Environment” (Kennedy, 2024)—a supportive socio-psychological space that sustains the dialogical self and turns development into an open-ended exploration of human possibilities.

### 4.3 The relational pedagogical paradigm: the ontological dissolution of adultcentrism and bleak pedagogy

By redefining growth as a non-linear, situational, and co-evolutionary flow, we revoke the adult’s self-appointed status as the teleological judge and disciplinary engineer of the child’s life. This paradigm functions by simultaneously deconstructing the AD-based worldview and its manifested BP practices across three dimensions: the reclaiming of temporal presentness, the restoration of intersubjective agency, and the creation of a “holding environment” that replaces the tyranny of norms.

#### 4.3.1 From deficit encoding to ontological presentness: dissolving adult dominance

To dismantle the adult-centric dominance inherent in Bleak Pedagogy, the Relational Pedagogical Paradigm replaces the deficit encoding of childhood with the principle of ontological presentness. By rejecting the Aristotelian reduction of the child to mere potency (dynamis), this framework affirms the child’s existence as a complete reality in the *aionian* present, thereby dissolving the pre-authorized mandate for adult intervention established in traditional teleological models.

By replacing the linear trajectory of becoming-adult with the subversive force of becoming-child (Deleuze and Guattari, 1980/1987), we argue that a child’s value is not a “future debt” to be paid upon reaching adulthood, but resides in the intensive flow of their current relational expression. Unlike the successive presents of *Chronos*—the disciplinary time that fuels BP’s “violence of the timetable” (Josselson, 1995)—*Aion* reclaims childhood as a site of “Virtual Mastery” (Kennedy, 2024).

This shift directly revokes the expert-patient model and the objectifying “I-i” relationship (Murris, 2016). When development is no longer a spatial displacement toward a predetermined adult *Telos*, the maturity gap ceases to be a legitimate ground for disproportionate authority. Adultcentrism is thus stripped of its ideological mask: the adult is no longer the teleological surveyor of a child’s lack, but a co-witness to the child’s Co-becoming. By affirming the ontological dignity of the “here and now,” the Relational Paradigm ensures that any pedagogical encounter is a meeting of two agents, effectively curing the ontological violence inherent in the repressive mechanisms of Bleak Pedagogy.

#### 4.3.2 From future-investment to intersubjective encounter: surpassing instrumentalism

The operational logic of Bleak Pedagogy (BP) relies heavily on the instrumentalism of future-investment. Under the adult-centric gaze, childhood is hollowed out and treated as a mere “transitional life form” (Kennedy, 2006), a raw material to be disciplined into a valuable future product. This “teleological temperament” (Xiong, 2008) justifies the “breaking of the child’s will” as a necessary pruning of agency that deviates from adult-predefined trajectories. The Relational Pedagogical Paradigm surpasses this instrumentalist plunder by grounding developmental dynamics in the “Shared Intentionality” of Tomasello (2019) and the “Co-becoming” framework of Lerner (2018).

By redefining the driver of development not as an isolated biological engine but as an evolved urge for collaborative negotiation, we expose the scientific fallacy of BP. If development is a “non-linear, temporal, and embodied process” (Gabriel, 2021), then the adult-centric attempt to engineer a standardized future adult is an ontological impossibility. We argue that any manifestation of a child’s agency is not an obstacle to development to be crushed, but the very site of shared Intentionality through which the self is constituted. Consequently, the “manipulative care” (Florio et al., 2022) inherent in BP is replaced by pedagogical presence—a state of being where the adult no longer acts as a proxy for the future, but as a partner in the present.

This shift deconstructs the adult-centric fear of life-heterogeneity. When education is restored as an encounter rather than a production project, the child is liberated from the alienated contract of surrendering current agency for future validation. By embracing “Relational Ontology” (Overton, 2015), the Relational Paradigm ensures that the teacher-child relationship is governed by mutual resonance and intersubjective negotiation, effectively terminating the violent logic that treats the child’s here and now as a mere functional sacrifice for an adult-defined *Telos*.

#### 4.3.3 From tyranny of norms to holding environment: dismantling corrective violence

The final operational pillar of BP is the Tyranny of Norms, a mechanism that weaponizes the evolutionary ladder of development to pathologize any deviation from the adult-centric mean. This tyranny utilizes the “violence of the timetable” (Josselson, 1995) to justify surveillance and corrective intervention, reducing the child’s heterogeneity to a deficit requiring repair. The Relational Pedagogical Paradigm dismantles this regime of measurement by applying Overton’s (2015) “Process-Relational Worldview” and the contextual turn, which redefine the educational site as a “Holding Environment” (Kennedy, 2024).

By shifting the focus from universal stage theories to “lifelong plasticity” (Lerner, 2002) and situational becoming, we expose the adult-centric bias inherent in standardized norms. If development is a dynamic fusion of person and multifaceted context, then the rigid yardstick of BP is not a scientific truth but a tool for “standardized social exclusion” (Vandenbroeck, 2006). In the Relational Paradigm, evaluation is transformed from deficit measurement into relational witnessing. Within the holding environment, the adult’s authority is no longer derived from expert superiority or the role of a disciplinary engineer, but from the reciprocal responsibility to sustain the child’s dialogical self.

This reconstruction ultimately transcends the adult-centric desire for correctivity and all-encompassing surveillance. By acknowledging that deviation is often a manifestation of situational creativity rather than a risk of derailment, the Relational Paradigm liberates the child from the fear of being diagnosed or repaired. In this space, the teacher-child relationship is governed by dialogical reciprocity, ensuring that education ceases to be a standardized production line and instead becomes a cradle of vital diversity, effectively dissolving the corrective violence that defines Bleak Pedagogy.

## 5 Conclusion

This paper has executed a meta-theoretical deconstruction and reconstruction of the concept of development, identifying it as the ideological epicenter of Adultcentrism that sustains the repressive practices of Bleak Pedagogy. By tracing the historical encoding of development from Aristotelian *Entelecheia* to modern linear evolution, we have exposed how the deficit conception of childhood provides a seemingly scientific sanctuary for the will-breaking and corrective violence inherent in BP.

The Ontological Inversion proposed in this study—shifting from prescribed ladders to a Process-Relational Becoming—offers more than a mere pedagogical alternative; it demands a fundamental reorientation of the adult-child encounter. By reclaiming the *Aionian* presentness of the child and establishing a Holding Environment based on shared intentionality, we strip adultcentrism of its teleological authority. The Relational Pedagogical Paradigm thus transforms education from a standardized production line into a site of intersubjective resonance.

Despite its theoretical contributions, this study remains primarily at the philosophical level; future empirical research is needed to investigate how this relational paradigm can be integrated into specific classroom settings and clinical practices. The significance of this research lies in its potential to liberate educational practice from the tyranny of norms. While biological immaturity remains a fact, the ways in which we interpret growth are matters of choice and power. Moving forward, the Relational Pedagogical Paradigm invites educators and researchers to view childhood not as a functional lack to be repaired, but as a unique state of human existence characterized by infinite creativity and situational becoming. In doing so, we do not merely facilitate development; we witness and participate in the flourishing of life itself.
