# Correction: The ontological inversion: transcending adultcentrism in the concept of development for the critical deconstruction of bleak pedagogy

**DOI:** 10.3389/fpsyg.2026.1938294

**Published:** 2026-08-03

**Authors:** Yao Luo, Xi Yang

**Affiliations:** School of Education, Hunan Normal University, Changsha, China

**Keywords:** adultcentrism, bleak pedagogy, development, intersubjectivity, ontological inversion, process-relational becoming

Author Yao Luo was erroneously included as corresponding author. The correct corresponding author is Xi Yang.

The original version of this article has been updated.

